# Rapid altitude displacement induce zebrafish appearing acute high altitude illness symptoms

**DOI:** 10.1016/j.heliyon.2024.e28429

**Published:** 2024-03-22

**Authors:** Jiahui Ma, Yilei Ma, Jia Yi, Pengyu Lei, Yimeng Fang, Lei Wang, Fan Liu, Li Luo, Kun Zhang, Libo Jin, Qinsi Yang, Da Sun, Chi Zhang, Dejun Wu

**Affiliations:** a Institute of Life Sciences & Biomedical Collaborative Innovation Center of Zhejiang Province, Wenzhou University, Wenzhou, 325000, China; b National and Local Joint Engineering Research Center of Ecological Treatment Technology of Urban Water Pollution, College of Life and Environmental Science, Wenzhou University, Wenzhou, 325035, China; c Wenzhou Institute, University of Chinese Academy of Sciences, Wenzhou, 325000, China; d Affiliated Dongguang Hospital, Southern Medical University, Dongguang, 523059, China; e Bioengineering College of Chongqing University, Chongqing, 400044, China; f Zhejiang Provincial Key Laboratory for Water Environment and Marine Biological Resources Protection, College of Life and Environmental Science, Wenzhou University, Wenzhou, 325000, China; g Department of Clinical Translational Research, The Third Affiliated Hospital of Wenzhou Medical University, Wenzhou, 325200, China; h Emergency Department, Quzhou People's Hospital, Quzhou, 324000, China

**Keywords:** High altitude environment, Acute high altitude illness, Zebrafish, Environmental simulation, Model construction

## Abstract

Rapid ascent to high-altitude areas above 2500 m often leads to acute high altitude illness (AHAI), posing significant health risks. Current models for AHAI research are limited in their ability to accurately simulate the high-altitude environment for drug screening. Addressing this gap, a novel static self-assembled water vacuum transparent chamber was developed to induce AHAI in zebrafish. This study identified 6000 m for 2 h as the optimal condition for AHAI induction in zebrafish. Under these conditions, notable behavioral changes including slow movement, abnormal exploration behavior and static behavior in the Novel tank test. Furthermore, this model demonstrated changes in oxidative stress-related markers included increased levels of malondialdehyde, decreased levels of glutathione, decreased activities of superoxide dismutase and catalase, and increased levels of inflammatory markers IL-6, IL-1β and TNF-α, and inflammatory cell infiltration and mild edema in the gill tissue, mirroring the clinical pathophysiology observed in AHAI patients. This innovative zebrafish model not only offers a more accurate representation of the high-altitude environment but also provides a high-throughput platform for AHAI drug discovery and pathogenesis research.

## Highlights

• Finding a new disease model was based on zebrafish.
• A better preclinical study subjects for acute high altitude illness (AHAI) was provided.
• A better high-throughput drug screening for AHAI was provided.
• A low-cost and flexible AHAI research equipment was established.

## 1 Introduction

With the burgeoning of tourism and economic activities in high-altitude areas, there has been a significant increase in the number of tourists or visitors from low-altitude areas who travel to high-altitude area. This sudden shift to high altitudes, typically abover 2500 m, expose individuals to hypobaric hypoxia, triggering acute high altitude illness (AHAI) [1,2]. AHAI encompasses a spectrum of conditions including acute mountain sickness (AMS), high-altitude cerebral edema (HACE), and high-altitude pulmonary edema (HAPE), each presenting distinct physiological challenges and health risks [[2], [3], [4], [5]]. The incidence of AMS, characterized by headache, burnout, dizziness, and nausea, can occur within 6–24 h of ascent and has been a subject of extensive study [6,7]. More severe forms, HACE and HAPE, may contribute to mortality rates as high as 40% in people with AHAI particularly in the areas that medical facilities are limited [8]. Not only does this have an impact on their daily lives, but it also poses significant challenges for health care workers and members of the military with special missions as well [9,10].

Traditionally, animal models such as mice, rats, and pigs have been used in pre-clinical studies to simulate high-altitude conditions in low-pressure chambers [[11], [12], [13]]. However, these models have limitations, including high costs, ethics for animal manipulation to reduce pain, a maximum of only 20 samples per session, and disruptions in the experimental conditions due to feeding requirements [14,15]. Moreover, these models often lack the capability for systematic behavioral assessments within the chamber and are burdened with long modeling times and waiting cycles. In light of this, it is urgently necessary to develop a novel device and an animal model that can better reflect the real situation in high-altitude areas for high-throughput testing of altitude sickness drugs with ideal efficacy and preventive function.

**Fig. 1:**
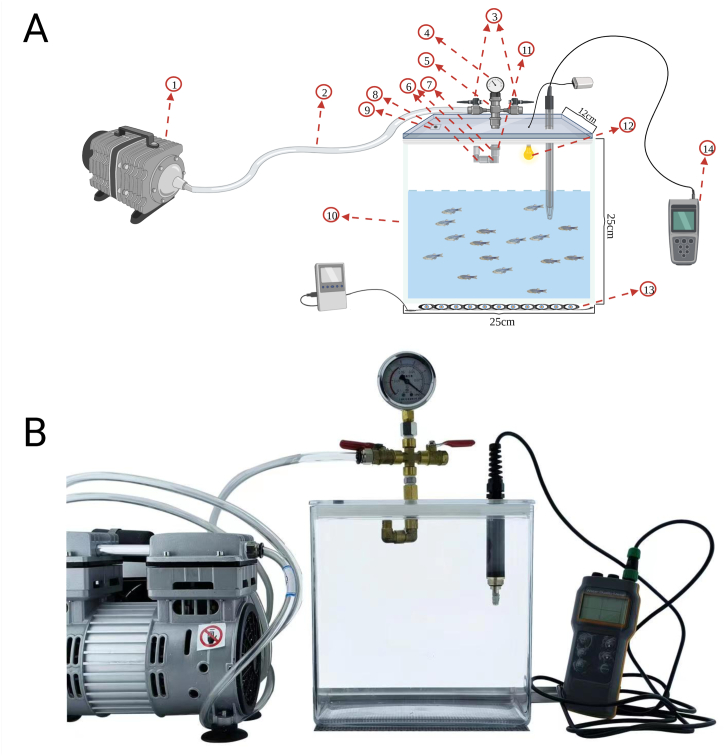
Self-assembled water vacuum transparent chamber equipment schematic, physical drawings and environmental simulation index test. (A) A schematic diagram of the device and its construction in this study are as followed: 1. vacuum pump; 2. plastic pipe; 3. small ball valve; 4. pressure gauge; 5. four-way pipe; 6. inside and outside wire elbow; 7. rubber washer; 8. Rubber check valve; 9. nano-adhesive; 10. 24 cm × 13 cm × 24 cm cuboid sink; 11. the extension pipe and the two-part wire 304 stainless steel pipe; 12. periodic controlled lighting system device; 13. hermally heated wire/semiconductor temperature control system equipment; 14. dissolved oxygen and temperature tester. (B) The physical picture of the equipment in this study.

The zebrafish model has the following advantages [[16], [17], [18], [19], [20], [21]]: Low economic cost, small organisms, large progeny production, linear homology of genes and high throughput. Furthermore, the preservation of immune system constituents in farmed zebrafish, coupled with the accessibility of transgenic lines that facilitate *in vivo* tracking of particular processes within a complete organism [22]. Recently, zebrafish has become a popular model to study many physiological and pathophysiological processes in humans, especially in the area of oxidative stress [[23], [24], [25]]. It is worth noting that the gills of zebrafish perform the same gas exchange function as mammalian airways, with a comparable structure featuring a respiratory epithelium coated in mucus, containing immune cells and smooth muscle cells located at the lamellar base. Additionally, the actions and functions of macrophages and neutrophils play a crucial role in lung inflammatory diseases and exhibit remarkable similarities between humans and zebrafish. A key experimental benefit is the direct exposure of gill tissue to ambient water, which enables direct targeting with waterborne substances without invasive procedures [[26], [27], [28]]. At the same time, zebrafish is also a classic animal model for studying blood-brain barrier integrity and local pathology (*e.g.* edema) *in vivo* [29,30]. Recognizing these advantages, a static self-assembled water vacuum transparent chamber equipment was designed to induce AHAI symptoms in zebrafish to provide a high-throughput animal model for future drug screening of AHAI (Fig. 1). We screened out the optimal conditions to induce the zebrafish AHAI model, and compared the physiological and pathological changes at the behavioral assessment, inflammatory factor level, oxidative stress level, and histological level with the clinical changes of AHAI patients to prove the feasibility of our model. The successful construction of this model is conducive to further research on the pathogenesis of AHAI, and provides an ideal platform for high-throughput screening of anti-AHAI drugs.

## 2 Materials and methods

### 2.1 Experimental animals

Wild-type AB adult zebrafish (1:1 male and female) (Zebrafish Resource Center, Wuhan, China), the adult zebrafish (4 months old) were reared in an aquatic animal breeding and reproduction system (Haisheng Marine Biological Equipment Co., Shanghai, China). The animal room light cycle was 14 h light and 10 h dark, and the temperature was maintained at 28.5 ± 1 °C. Zebrafish in each group were placed in the static self-assembled water vacuum transparent chamber equipment. The control group was not treated, and the experimental group was set with pressure values corresponding to the actual altitude, and different induction duration was regulated. The barometric pressure drop to the specified value was completed 10 min after the vacuum pump was started.

### 2.2 Experimental design and sample collection

The construction and physical picture of zebrafish AHAI model building equipment was described in the legend of Fig. 1A and B. 20 farmed zebrafish were randomly selected and placed in the corresponding equipment of each group for 15 days for domestication (not take the factor of sex into account). After the end of the adaptation period, the control group remained unchanged, and the other two groups were treated with a 2 L silent oil-free vacuum pump (JBL-1100 W, Zhejiang Fujii Air Compressor Co., Ltd., Zhejiang, China) (pumping rate = 30 L/min) make the barometric pressure in the equipment reach the corresponding barometric pressure in the high-altitude area after 3 days at 3500 m, 4 days at 5000 m, 2 days at 6000 m, 10 h at 5000 m, 5 h at 6000 m and 6 h at 6000 m (shown as 3500 m 3d, 5000 m 4d, 5000 m 2d, 5000 m 10h, HA 5h and HA 2h, respectively), and record the temperature and dissolved oxygen content. Immediately after the modeling was finished, the zebrafish were euthanised at ultra-low temperature (in liquid nitrogen) to ensure the rapid cessation of life activities or conduct behavioral evaluation. Zebrafishes were killed at ultra-low temperature and cut off by surgical scissors from the heart to the side to preserve the head (hereafter referred to as the collected head tissue), which contains the heart, brain and gill directly related to the effects of AHAI. In a word, the head was collected, weighed and transferred to the −80 °C refrigerator (YC-80, Aucma Co., Ltd., Shanghai, China) for further analysis. Here, by measuring the above modeling conditions, for indicators of inflammation, 8 zebrafish in each group were selected as actual samples to determine the relevant biochemical parameters including interleukin-1β (IL-1β), interleukin-6 (IL-6) and tumor necrosis factor-α (TNF-α). Meanwhile, for oxidative stress-related indicators, after randomly allocated the collected heads to 8 samples at 0.1 g, follow-up tests were performed for malondialdehyde (MDA), glutathione (GSH) and catalase (CAT), superoxide dismutase (SOD) and reactive oxygen species (ROS), which were determined to verify whether the model conforms to clinical indications and is feasible.

### 2.3 Behavioral testing

Behavioral tests were evaluated using the novel tank test (NTT), slightly modified from the methodology described by Quadros et al. [31]. NTT exploits zebrafish's instinctive behavior of seeking shelter when exposed to an unfamiliar environment. Under stress, zebrafish tend to reduce exploratory behavior, so we chose total distance, average speed, top, and freezing to evaluate possible behavioral anomalies in zebrafish under the AHAI conditions [32,33]. In addition, spending long periods of time at the bottom of the tank can reflect anxiety-like behavior in zebrafish [[34], [35], [36]]. Thus, NTT is feasible as an indicator of changes in AMS behavior. In short, NTT is carried out directly in the experimental equipment (5.2 L, 24 height × 24 length × 13 width, cm), basically divided into upper and lower halves. Behavioral tests were conducted to establish the frequency of the top transition, time spent at the top, and delay in entering the top area in the NTT. Six cameras were utilized to capture the behavior tests over a 5-min period. The experiment was conducted three times. Fish Track (zebrafish analysis software, Shanghai Xinsoft Information Technology Co., Ltd., Shanghai, China) was used for analysis.

### 2.4 Head biochemical analyses

After the collected head tissue was removed from the −80 °C refrigerator, it was homogenized in 0.9% physiological saline (1:9 [wt/wt]), and subsequently centrifuged at 4 °C and 3000 × rpm for 20 min, and the resulting supernatant was acquired for biochemical scrutiny (The weight of each head is shown in Table S1). The levels of IL-1β, IL-6 and TNF-α in the head were detected by ELISA kit (ZIKE Biotech, Shenzhen, China) according to the instructions of the manufacturer.

### 2.5 Flow cytometry (FCM) for reactive oxygen species content detection

After 2 h of induction with the equipment at 6000 m, the tested animals were killed immediately. The collected head tissues were cut off and retained only brain tissues, gill tissues and heart were, and taken 0.1 g tissue as a sample. The tested animals were cleaned and repeated 3 times with PBS.

Digestive enzymes in the Tissue digestion kit (KGA829, Jiangsu KeyGEN Biotechnology Co., Ltd., Jiangsu, China) prepared with KeyGEN cell suspension were digested in a 120 × rpm constant temperature shaker at 37 °C in the dark for 2 h; The digestive enzyme solution were filtered with a 200-mesh sieve, cell suspensions were retained, ROS was labeled with a DCFH-DA fluorescent probe (S0033S, Shanghai Beyotime Biotechnology Co., Ltd., Shanghai, China), and intracellular ROS fluorescence intensity was detected and quantified by FCM.

### 2.6 Detection of oxidative stress related indexes

After the collected head tissue was removed from the −80 °C refrigerator, the homogenate was mixed with 1 mL of the corresponding extraction solution for every 0.1 g tissue, and centrifuged at 4 °C and 8000×*g* for 10 min. The supernatant was collected and processed according to the relevant instruction (Solarbio Biotechnology Co., Ltd., Beijing, China). The contents of MDA and reduced GSH, as well as the activities of SOD and CAT were measured and quantified.

### 2.7 H&E staining and pathological analysis of sections of brain tissue and gill tissue

The operation of slicing is slightly modified from the methodology described by Oliveira-Lima et al., [37]. Zebrafish brain tissue and gill tissue were taken after modeling, with light movements to avoid changes in the tissues. The tissues were fixed in 4% paraformaldehyde for 24 h, dehydrated with anhydrous ethanol gradient, made transparent with xylene, then soaked with soft wax and hard wax (G1128, Servicebio, Wuhan, China) at 65 °C. Subsequently, the tissues were removed the tissue and put in the hole of the encasing box, then filled with soft wax, carefully removed from the water bath after adjusting the tissue to a proper position. After the soft wax solidified, the soft wax was sealed and packed into the embedding box, and stored in the refrigerator at 4 °C. Slices were sliced with pathology slicer (RM2016, Shanghai Leica Instrument Co., Ltd., Shanghai, China), unfolded at 45 °C in tissue spreader (KD-P, Zhejiang Kehua Instrument Co., Ltd., Zhejiang, China), and then dried naturally with adhesive slides (G6012-1, Servicebio, Wuhan, China). The tissues were then dewaxed and rehydrated, followed by staining with hematoxylin dye solution (Harris) (G1076, Servicebio, Wuhan, China) for 3–5 min. They were washed with tap water for 1–2 min and subsequently differentiated in 1% hydrochloric acid alcohol solution for a few seconds to half a minute (99 ml 75% alcohol + 1 ml concentrated hydrochloric acid), until the color separated and the slice appeared pink, at which point they were removed immediately. The slices were rinsed with running water and returned to blue for 5–10 min and then shaken dry, then put in eosin dye solution (water-soluble) for 30–60 s and washed. This was followed by toning dehydration with 80% and 95% ethanol for 90 s; finally, the slices were immersed in anhydrous alcohol I and II for 1–2 min and in xylene I and II for transparency for another 1–2 min. The pathological condition was observed using an upright optical microscope (NIKON ECLIPSE E100, Nikon, Shanghai, China) after the slide was sealed with neutral gum (10004160, Sinopharm Chemical Reagent co., Ltd., Shanghai, China).

### 2.8 Statistics analyses and reproducibility

All statistical analyses were performed by using GraphPad Prism (GraphPad Software Inc. San Diego, California, USA). All data are expressed as means ± SEM except for box-and-whisker plots indicating the median. Significance was determined using a two-tailed unpaired *t*-test in levels of TNF-α, IL-6, IL-1β, SOD, ROS and behavioral indicators, ordinary one-way analysis of variance (ANOVA) in levels of CAT, MDA and GSH, and significance codes in Figures are as follows: *p < 0.05, **p < 0.01, ***p < 0.001, ****p < 0.0001. Recognizing the high degree of variability between groups in animal experiments, individual values are shown in the graph as dots of varying colours. In addition, the n values indicate the total number of validated zebrafish in each group. Because of the importance of repro-ducibility in biomedical studies [20], all experiments were repeated 3 times independently by 3 different researchers.

## 3 Result

### 3.1 Design of a self-assembling equipment for restoring high altitude environment and determining the optimum molding conditions

**Fig. 2:**
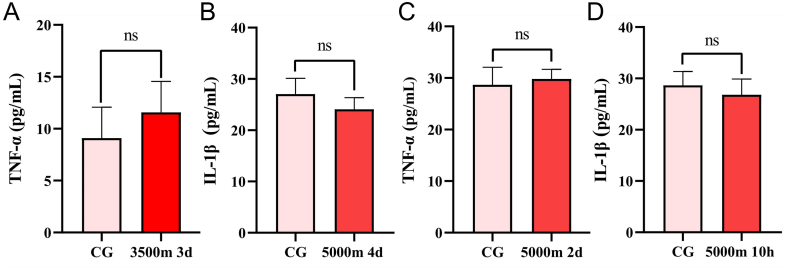
Proinflammatory cytokines of the collected head tissue containing brain, gills and heart in different stages. There were no statistically significant distinctions between the experimental and control groups (shown as CG) in (A), (B), (C), and (D) within the aforementioned conditions. Differences between these two groups were calculated for each transcript by the *t*-test. Significance was defined as ns *p* > 0.05 (ns, statistically non-significant) vs. control groups (n = 8 per group).

A self-assembled water vacuum transparent chamber equipment, designed and fabricated for restoring high altitude environment is shown in Fig. 1. The equipment is well-airtight and that the container is completely transparent. The pneumatic valve allows the barometric pressure inside the container to achieve a slow drop (*i.e.* a slow rise in altitude at which the zebrafish are located). The barometric pressure and dissolved oxygen levels in the experimental and control groups could be detected by means of a pressure gauge (YN60, Shanghai Lian Li Instrumentation Co., Ltd., Shanghai, China) and a dissolved oxygen detector (AZ86031, Taiwan Hengxin Technology Co., Ltd., Taiwan, China). The results of preliminary modeling conditions were found that the induction of 3500 m 3d, 5000 m 4d, 5000 m 2d and 5000 m 10h did not show any significant difference in TNF-α or IL-1β levels from those of the control group (Fig. 2A, B, C and D) while HA 2h group exhibited elevated levels of IL-6, IL-1β, and TNF-α compared to the control group (Fig. 5A and B and C). At the same time, the levels of CAT, GSH and MDA were not significantly different in the HA 5h group compared to the control group (Fig. 5A and B and C), which further proves the credibility of HA 2h as a molding condition. It is suggested that zebrafish may have adapted to the environment and returned to normal physiological activities after 5 h at 6000 m. Additionally, the acute stress response after 2 h at 6000 m was also studied (barometric value, temperature and dissolved oxygen values are shown in Table 1).

**Table 1:** The measurement values of temperature, air pressure, and dissolved oxygen content were monitored by the equipment during the experiment of the control group and HA 2h group.

Grouping	Control group	HA 2h
Barometric pressure value (mbar)	1.0	0.4
Temperature (^o^C)	27.6	21.9
Dissolved oxygen (mg/L)	8.2	1.8

### 3.2 Zebrafish exhibited motor retardation

**Fig. 3:**
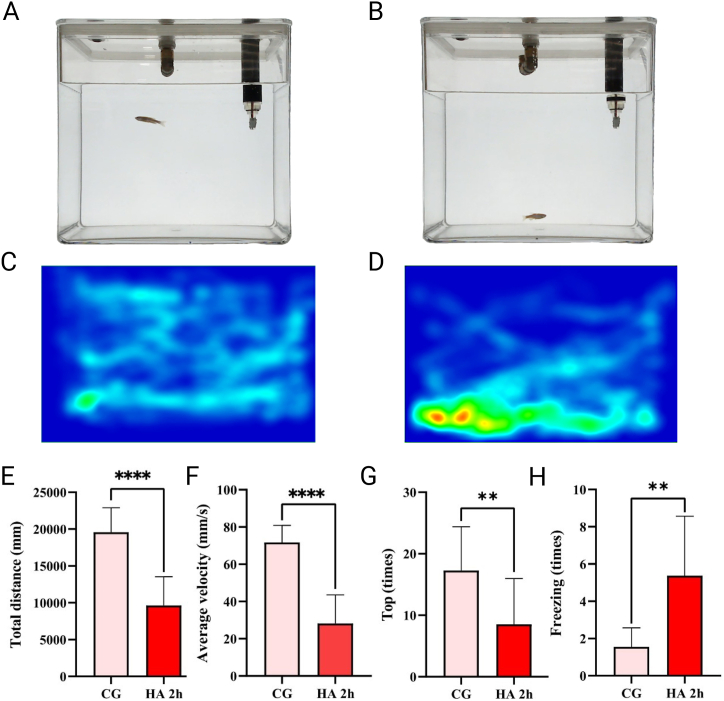
NTT behavioral evaluation of zebrafish in a simulated environment of after 2 h at 6000 m (shown as HA 2h). (A) NTT experiment picture display of control group (shown as CG). (B) NTT experiment picture display of HA 2h group. (C) NTT representative 2D thermal map for the control group. (D) NTT representative 2D thermal map for the HA 2h group. (E) In comparison to the control group, the total distance travelled by zebrafish in the experimental group demonstrated a significant decrease (*p* < 0.0001). (F) The average velocity of zebrafish in the experimental group decreased significantly (*p* < 0.0001). (G) The number of zebrafish entering the top decreased significantly in the experimental group (*p* = 0.0038). (H). The freezing times of zebrafish in the experimental group increased significantly (*p* = 0.0036). Differences between these two groups were calculated for each transcript by the *t*-test. Significance was defined as **p < 0.01, and ****p < 0.0001, vs. control groups (*n* = 8 per group).

After preliminary exploration determined that the simulated altitude of 6000 m and induction time of 2h may be the ideal conditions for building zebrafish AHAI model, NTT behavior test was used to further verify the rationality of the conditions. NTT behavioral tests were performed to determine whether zebrafish experience vertigo symptoms similar to humans (Fig. 3A and B) and the representative 2D thermal map of NTT is shown in Fig. 3C and D. See Video S1 and Video S2 for representative examples. In the HA 2h group, the overall distance and mean velocity were notably lower when compared to the control group (Fig. 3E and F), *i.e.* showing a significant reduction in locomotor activity, while the significant reduction in the times of trips to the top was the evidence of abnormal exploratory behavior (Fig. 3, G). In the meantime, remarkable increase in the freezing times (Fig. 3, H) indicated that the zebrafish are exhibiting symptoms similar to weakness. Above results was consistent with the clinical symptoms of vertigo accompanying patients with AHAI.

### 3.3 Increased levels of oxidative stress in the head of zebrafish

**Fig. 4:**
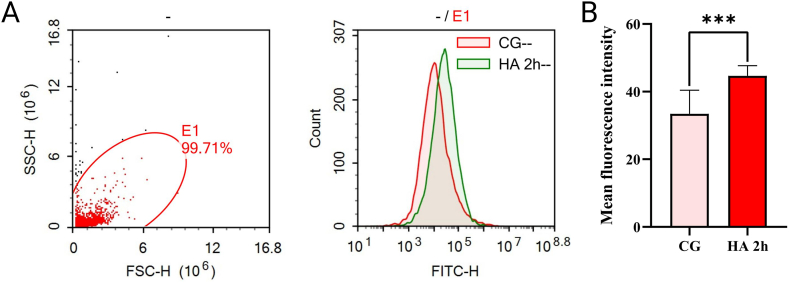
Detection of reactive oxygen species (ROS) in the tissue containing brain, gills and heart of zebrafish in the HA 2h group compared to the control group (shown as CG). (A). ROS stained with DCFH-DA probe was collected *via* Flow cytometry, and the result was counted as (B). Differences between these two groups were calculated for each transcript by the *t*-test. Significance was defined as ***p < 0.001, vs. control groups (*n* = 8 per group).

**Fig. 5:**
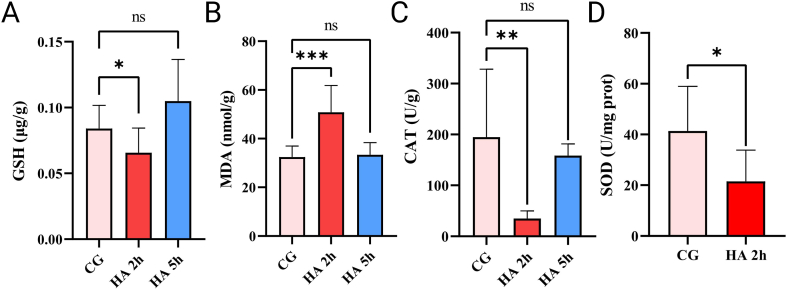
Oxidative stress level of zebrafish use the head containing brain, gills and heart in the, HA 5h group, HA 2h group and control group (shown as CG). (A) GSH. GSH levels in HA 2h group were lower than those in the control group (*p* = 0.0260)., while there was no significant difference in HA 5h group (p = 0.1470). (B) MDA. HA stimulation of MDA secretion in comparison with control group (*p* = 0.0311), while HA 5h group had no significant difference (p = 0.7570). (C) CAT. Compare with control group, HA decreased CAT activity (*p* = 0.0036), while HA 5h group had no significant difference (p = 0.5638). (D) SOD. Compare with control group, HA decreased SOD activity (*p* = 0.0206). The data of Fig. 5A–C were expressed as mean ± S.E.M. and analyzed using analysis of variance (one-way ANOVA) followed by the Tukey's post hoc test and the differences of Fig. 5D between these two groups were calculated for each transcript by the *t*-test. Significance was defined as *p < 0.05, **p < 0.01, ***p < 0.001, and ns > 0.5, vs. controls groups (*n* = 8 per group).

To further demonstrate the success of the device in enabling zebrafish to develop AHAI, markers of oxidative stress associated with changes in humans after AHAI were examined. In tests performed on the head after the construction of the zebrafish AHAI model after 2 h at 6000 m (HA 2h) groups and after 5 h at 6000 m (HA 5h) groups respectively, we found a highly significant increase in the levels of ROS (Fig. 4A and B) and MDA (Fig. 5, B) in the HA 2h group compared to the control group, as well as a significant decrease in the levels of SOD, CAT, and GSH (Fig. 5A–C, and D), which was attributed to the fact that low-pressure hypoxia decreases the activity of the antioxidant enzyme system, thus leading to oxidative stress [[38], [39], [40]].

### 3.4 Inflammation in the head of the zebrafish

**Fig. 6:**
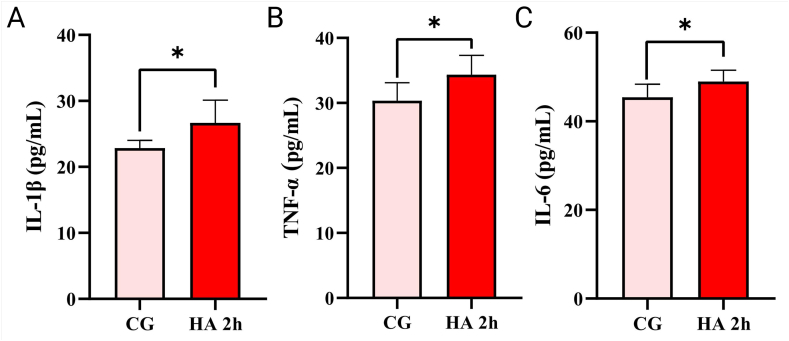
Proinflammatory cytokines of zebrafish use the head containing brain, gills and heart in the HA 2h group compared to the control group (shown as CG). (A) IL-1β levels were elevated in the HA group compared to the control group (p = 0.0260). (B) TNF-α. Compare with control group, HA promoted TNF-α secretion (p = 0.0146). (C) IL-6. Compare with control group, HA promoted IL-6 secretion (p = 0.0206). Differences between these two groups were calculated for each transcript by the *t*-test. Significance was defined as *p < 0.05, vs. control groups (n = 8 per group).

AHAI induces oxidative stress while also triggering inflammation, which deranges the levels of pro-inflammatory cytokines [39,41,42]. Firstly, 3500 m for 3 d, 5000 m for 2 d, 5000 m for 4 d, and 5000 m for 10 h environmental conditions were induced and examined TNF-α and IL-1β levels, respectively, while continuing to induce good modeling parameters for HA 2h based on previous studies. After the construction of the zebrafish AHAI model, the heads were taken and tested for the pro-inflammatory cytokines IL-6, IL-1β and TNF-α. It was found that the HA 2h group exhibited higher levels of IL-6, IL-1β and TNF-α than the control group (Fig. 6A and B and C), which may be due to the persistence of ROS and the large release of inflammatory mediators caused by neurohumoral and hemodynamic responses [43,44].

### 3.5 Edema and inflammatory cells infiltration in the gills of zebrafish in a high altitude area

**Fig. 7:**
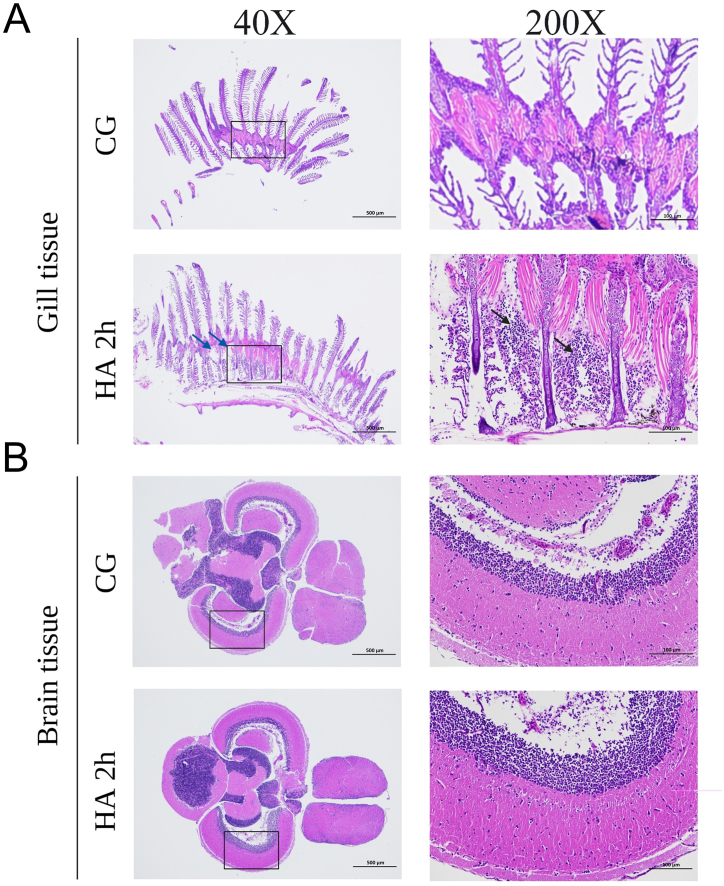
Histologically assessed H&E stained sections of brain tissue and gill tissue from the HA 2h group compared to the control group (shown as CG). (A) Gill filaments extended from the convex surface of the gill arch, and the other end was free, many small semi-circular flat sacc-like branchial flakes extend to both sides of the branchial filaments, which are evenly distributed perpendicular to the branchial filaments and contain abundant capillaries without obvious abnormalities in the control group. The HA 2h group can be obviously observed massive inflammatory cell invasion (black arrow) and very slight edema (blue arrow). (B) The number of neurons in the brain tissue was abundant and closely arranged and the morphology and structure of neurons were normal, as well as no obvious abnormalities were found.

AHAI leads to an increase in vascular permeability, resulting in mild edema, which may become further severe during progression. As shown by H&E staining, no obvious abnormalities were observed in control group gill tissue. However, there are more inflammatory cell infiltration in the tissues (black arrow) and very slight edema signs (blue arrow) in HA 2h group (Fig. 7A). Meanwhile, the number of neurons in the brain tissue was abundant and closely arranged. The morphology and structure of neurons were normal, and no obvious abnormalities were found in CG or HA 2h group (Fig. 7B). These results indicated that the respiratory system of zebrafish has been affected, which may be the main cause of the AHAI-like symptoms.

## 4 Discussion

Acute high altitude illness (AHAI) is a physiological response that occurs in unacclimatized individuals following a rapid ascent to an altitude of 2500 m or higher. It is generally delayed by 4–12 h and develops at any time within 5 days. Acute mountain sickness (AMS) (particularly above 4000 m, where the incidence rate can reach 40–90 percent) can proceed to high-altitude pulmonary edema (HAPE) and high-altitude cerebral edema (HACE), which are further life-threatening [45,46].

To address the massive flaws in modeling or screening drugs such as mice and rats, AHAI in zebrafish model animal was researched, which have a comparable blood-brain barrier, strong genetic similarities and respiratory metabolism to humans [[26], [27], [28], [29], [30]]. At the same time, zebrafish respond to many pathological factors with robust oxidative stress [47]. In addition to restoring high-altitude environmental conditions with the equipment, direct behavioral evaluation can be carried out after modeling, taking full use of the advantages of high throughput zebrafish. According to previous studies, the corresponding barometric pressure changes by 0.1201 mbar for every 1 m change in altitude, and the oxygen partial pressure and temperature also change simultaneously [48,49]. Therefore, firstly, the optimal conditions were explored and determined by inducing molding conditions with different altitudes and residence times, and using indicators related to inflammation and oxidative stress. The results showed that after the residence time of more than 5 h, the relevant indicators of zebrafish head were not significantly different from those of the control group, but the residence time of 2 h was significantly different The oxidative stress recovery after 5h at 6000 m seen in this study is similar to the mechanism by which human acute exposure and adaptation to high altitude *in vivo* may restore REDOX homeostasis by triggering an adaptive response to oxidative stress. Meanwhile, ctivation of the enzyme's antioxidant defenses was able to reduce oxidative stress biomarkers to pre-exposure levels after a period of time [50]. Therefore, this condition was determined to be the most suitable condition for inducing zebrafish AHAI model and conducted follow-up experiments. Anoxic survival of fish needs to be achieved by regulating the blood oxygen affinity to increase the tissue's ability to absorb and transport oxygen or by reducing oxygen consumption through strong metabolic rate inhibition [51]. In previous studies, Zebrafish's model of acute anoxia was conducted by anoxia (1% O_2_ and 99% N_2_) for 2 h [52], which is consistent with the conditions explored in this study.

In the novel tank test (NTT) behavioral test, zebrafish exhibited slow movement, abnormal exploration behavior and static behavior that proved the reliability and reproducibility of the AHAI model. Almost everyone who climbs to high-altitude above 4500 m experiences headache, weariness, dizziness, or vertigo [53]. In addition, previous research has also demonstrated that AHAI significantly affects both locomotor activity and rotarod behavior tests in mice [54], and the findings in this study are consistent with above experiment. It is suggested that this model may effectively restore the high altitude environment and make the tested zebrafish show the pathological state similar to AHAI.

After demonstrating significant changes in behavior, it is important and necessary to evaluate physiological and pathological changes related to AHAI *in vivo*. reactive oxygen species (ROS) are tiny, unstable molecules created by oxygen (O_2_) [55]. Oxidative stress arises when the body undergoes heightened environmental and chemical effects that cannot be counteracted by its endogenous antioxidant mechanisms, including superoxide dismutase (SOD), catalase (CAT), and glutathione (GSH) [11,56]. During the progression of AHAI, the oxidative stress biomarkers ROS and lipid oxidative damage (as well as malondialdehyde (MDA) levels) will increase, as well as GSH levels and SOD and CAT activities of endogenous antioxidant system members will decrease due to decreased oxygen partial pressure at each point in the oxygen transport cascade of acute mitochondria [40,57]. In this study, ROS levels in the model caused by 6000 m for 2 h increased significantly. Meanwhile, in the control group, HA 2h group and HA 5h group, a substantial increase was found in MDA levels after 2 h of induction, and the CAT activities and GSH levels declined dramatically, which is consistent with oxidative stress-related changes in AHAI patients in previous studies [[58], [59], [60]]. Furthermore, SOD levels were lowered within 2 h after induction, indicating that zebrafish may adjust to the high-altitude environment faster than mammals, a novel insight that could have significant implications for understanding AHAI. 6000 m for 2 h is probably a plausible environment for the induction of the AHAI model in zebrafish, which is a key parameter.

Follow-up experiments were carried out at a predetermined high-altitude induction of 6000 m for 2 h. Elevated oxidative stress may upregulate the nuclear factor kappa-B (NF-κB) since increased levels of NF-κB were found in the brains of rats subjected to acute hypoxia. Following that, NF-κB is a regulator of pro-inflammatory cytokine expression (IL-6, IL-1β and TNF-α) [20]. In the rat brain, it causes inflammatory reactions and promotes transvascular leakage [61]. The results demonstrated that IL-6, IL-1β, and TNF-α levels rose after 2 h of induction at high-altitude, which was consistent with relevant findings [62]. It indicates that zebrafish likewise showed reduced inflammation at an altitude of 6000 m for 2 h [9]. Prior to this, there was no significant increase in inflammatory indicators in the different high-altitude environments simulated, further confirming that 6000 m for 2 h might be a suitable condition for the zebrafish AHAI model.

On the other hand, free radicals can damage endothelial cells in the blood-brain barrier, resulting in extracellular edema and inflammatory responses, such as those seen in AMS and HACE [61]. Simultaneously, a decrease in oxygen partial pressure results in decreased ventilation and interstitial pulmonary edema, as well as reduces the oxygen supply in the brain, increases cerebral blood flow of up to 26%, resulting in cerebral edema, breakdown of the blood-brain barrier, and other complications [63,64]. In this study, it was found that obvious inflammatory cell infiltration and slight edema appeared in the gill tissue after acute altitude exposure, which was consistent with clinical symptoms. No obvious abnormal signs were observed in the brain tissue, suggesting that the disease may not have progressed to the HACE stage. Indeed, clinical data showed that brain Edema in the AMS stage is not obvious [9,65,66]. Additionally, based on a comparison of the device model developed in this study with the indicators applied in building the AHAI model for human, mouse, and rat subjects (as illustrated in Table 2). It can be concluded that the zebrafish AHAI model is basically consistent with the clinical and existing animal models in terms of biochemical indicators of inflammation, biochemical indicators related to oxidative stress, abnormal behavior, and histological changes, which can further provide conditions for disease control and drug screening in the future.

**Table 2:** Modeling conditions and related detection results of various experimental subjects and zebrafish AHAI disease model construction in this study.

Experimental subject	Human	Mice	Rat		Zebrafish
**Experiment condition**	3200 m–3800 m 24 h or 48 h Designated altitude area	7000 m–8000 m 12 h, 24 h or 70 h animal decompression chamber	3860 m–4200 m 2 d animal decompression chamber	6000 m–8000 m 9 h-3 d animal decompression chamber	6000 m 2 h self-assembled water vacuum transparent chamber equipment
**Inflammation-related biochemical indicators**	IL-6, IL-1β, TNF-α↑	IL-1β, TNF-α, IL-6↑	TNF-α, IL-1β, IL-6↑	IL-1β, IL-6, TNF-α↑	IL-1β, IL-6, TNF-α↑
**Biochemical indicators related to oxidative stress**	MDA↑, SOD↓	GSH, SOD, CAT↓; MDA↑,ROS↑	SOD, GSH↓; MDA↑	SOD, GSH, CAT↓; MDA, ROS↑	SOD, GSH, CAT↓; MDA, ROS↑
**Abnormal behavior**	Dizziness, fatigue, headache	**/**	Impairment of learning, memory function and cognitive function	Impairment of learning, memory function and cognitive function. Deficits in spatial memory and exploratory behavior	slow movement, abnormal exploration behavior and static behavior
**Tissue state**	**/**	**/**	**/**	Pulmonary edema, thickening of alveolar walls and alveolar septum, bleeding, a large number of inflammatory cells and red blood cells dispersed.	Extensive inflammatory cell infiltration and slight edema in the gill
**Ref.**	[61,[67], [68], [69]]	[60,70,71]	[61,72]	[59,[73], [74], [75], [76], [77], [78]]	This study

Finally, it should be emphasized that the equipment is quite adaptable. It may be transformed into a device for analyzing relevant behavioral indications (recording movement routes) from a top perspective by simply modifying the location of the pressure gauge connection system, enhancing its utility in behavioral studies. Despite these advancements, the study has limitations. Further research is needed to explore gene-level changes associated with AHAI, the applicability of different altitude settings, and the suitability of this model for zebrafish larvae. Additionally, due to resource constraints, the study did not investigate changes at the pulmonary system level, which is crucial for a comprehensive understanding of AHAI. Future studies should aim to bridge these gaps, aligning more closely with clinical symptoms and providing a more holistic view of AHAI pathophysiology.

## 5 Conclusion

In conclusion, this study successfully developed a novel zebrafish-based model for acute high altitude illness research, validated through comprehensive behavioral, biochemical, and pathological analyses.This model addresses the limitations of traditional mammalian models, such as direct behavioral analysis, feeding convenience, and small sample sizes. The high-throughput nature of the zebrafish model positions it as a promising tool for future acute high altitude illness drug discovery and research.

## Ethics statement

The animal study was reviewed and approved by the Wenzhou University Committee.

## Data availability statement

The original contributions presented in this study are included in the article/supplemental material, further inquiries can be directed to the corresponding author.

## CRediT authorship contribution statement

**Jiahui Ma:** Writing – original draft, Investigation. **Yilei Ma:** Formal analysis. **Jia Yi:** Methodology. **Pengyu Lei:** Conceptualization. **Yimeng Fang:** Visualization. **Lei Wang:** Data curation. **Fan Liu:** Project administration. **Li Luo:** Resources. **Kun Zhang:** Resources. **Libo Jin:** Supervision. **Qinsi Yang:** Supervision, Software, Funding acquisition. **Da Sun:** Writing – review & editing, Validation, Funding acquisition. **Chi Zhang:** Resources. **Dejun Wu:** Writing – review & editing, Funding acquisition.

## Declaration of competing interest

The authors declare that they have no known competing financial interests or personal relationships that could have appeared to influence the work reported in this paper.

## Abbreviation

(AHAI): Acute high-altitude illness
(AMS): Acute mountain sickness
(CAT): Catalase
(FCM): Flow cytometry
(GSH): Glutathione
(HACE): High-altitude cerebral edema
(HAPE): High-altitude pulmonary edema
(IL-1β): Interleukin-1β
(IL-6): Interleukin-6
(MDA): Malondialdehyde
(NF-κB): Nuclear factor kappa-B
(NTT): Novel tank test
(ROS): Reactive oxygen species
(SOD): Superoxide dismutase
(TNF-α): Tumor necrosis factor-α
