# Supplementary material for: Rapid altitude displacement induce zebrafish appearing acute high altitude illness symptoms
Source: Heliyon. 2024 Mar 22;10(7):e28429. doi: 10.1016/j.heliyon.2024.e28429 (PMC10999933; doi:10.1016/j.heliyon.2024.e28429)
Supplement: Multimedia component 1 [file mmc1.docx]

Supplementary Material

**High altitude environment induce zebrafish appearing acute high altitude illness symptoms**

**Jiahui Ma ^1†^,** **Yilei Ma ^1†^, Jia Yi ^1†^, Pengyu Lei ^1^, Yimeng Fang ^1^, Lei Wang ^1^, Fan Liu ^1^, Li Luo ^2^, Kun Zhang ^3^, Qinsi Yang ^4^, Libo Jin ^5*^, Luqing He ^6*^ and Da Sun ^1*^**

^1^Institute of Life Sciences & Biomedical Collaborative Innovation Center of Zhejiang Province, Wenzhou University, Wenzhou 325035, China

^2^Affiliated Dongguan Hospital, Southern Medical University, Dongguan 523059, China

^3^Bioengineering College of Chongqing University, Chongqing 400044, China

^4^Wenzhou Institute, University of Chinese Academy of Sciences, Wenzhou 325000, China

^5^National and Local Joint Engineering Research Center of Ecological Treatment Technology of Urban Water Pollution, Wenzhou University, Wenzhou 325035, China

^6^Department of Science and Education, the Third People's Hospital Health Care Group of Cixi, Ningbo 315300, China

*** Correspondence:**Libo Jin, 20160121@wzu.edu.cn; Luqing He, helq619@163.com; Da Sun, sunday@wzu.edu.cn.

# Supplementary Data

Table S1 The weight of zebrafishes collected head in each group (Inflammatory index).

| **Experimental section** | **Weight** | | | | | | | | |
| --- | --- | --- | --- | --- | --- | --- | --- | --- | --- |
|  | **Sample (g)** | **1** | **2** | **3** | **4** | **5** | **6** | **7** | **8** |
| **IL-1β** | CG | 0.0456 | 0.0284 | 0.0407 | 0.0381 | 0.0522 | 0.0422 | 0.0702 | 0.0310 |
|  | 5000m 4d | 0.0672 | 0.0428 | 0.0411 | 0.0642 | 0.0532 | 0.0759 | 0.0351 | 0.0598 |
|  | CG | 0.0456 | 0.0452 | 0.0432 | 0.0441 | 0.0419 | 0.0305 | 0.0577 | 0.0279 |
|  | 5000m 10h | 0.0665 | 0.0652 | 0.0430 | 0.0437 | 0.0547 | 0.0318 | 0.0620 | 0.0317 |
|  | CG | 0.0330 | 0.0380 | 0.0578 | 0.0376 | 0.0573 | 0.0735 | 0.0793 | 0.0992 |
|  | HA 2h | 0.0267 | 0.0358 | 0.0381 | 0.0543 | 0.0583 | 0.0553 | 0.0431 | 0.0393 |
| **TNF-α** | CG | 0.0480 | 0.0672 | 0.0620 | 0.0846 | 0.0574 | 0.0668 | 0.0894 | 0.0478 |
|  | 3500m 3d | 0.0680 | 0.0485 | 0.0503 | 0.0460 | 0.0540 | 0.0820 | 0.0534 | 0.0623 |
|  | CG | 0.0310 | 0.0417 | 0.0535 | 0.0525 | 0.0520 | 0.0595 | 0.0493 | 0.0466 |
|  | 5000m 2d | 0.0383 | 0.0424 | 0.0369 | 0.0564 | 0.0524 | 0.0571 | 0.0563 | 0.0402 |
|  | CG | 0.0330 | 0.0380 | 0.0578 | 0.0376 | 0.0573 | 0.0735 | 0.0793 | 0.0992 |
|  | HA 2h | 0.0267 | 0.0358 | 0.0381 | 0.0543 | 0.0583 | 0.0553 | 0.0431 | 0.0393 |
| **IL-6** | CG | 0.0330 | 0.0380 | 0.0578 | 0.0376 | 0.0573 | 0.0735 | 0.0793 | 0.0992 |
|  | HA 2h | 0.0267 | 0.0358 | 0.0381 | 0.0543 | 0.0583 | 0.0553 | 0.0431 | 0.0393 |

**Video S1.** NTT representative example for the control group.

**Video S2.** NTT representative example for the HA 2h group.
